# Association of lipid-lowering drugs with risk of sarcopenia: a drug target mendelian randomization study and meta-analysis

**DOI:** 10.1186/s40246-024-00643-3

**Published:** 2024-07-03

**Authors:** Jiaxin Li, Chenyang Zang, Hui Lv, Zheng Xiao, Peihong Li, Bo Xiao, Luo Zhou

**Affiliations:** 1 Department of Neurology, Xiangya Hospital, Central South University, Changsha, Hunan China; 2 National Clinical Research Center for Geriatric Disorders, Xiangya Hospital, Central South University, Changsha, Hunan China; 3 Department of Dermatology, Xiangya Hospital, Central South University, Changsha, Hunan China; 4 Department of Pathology, First Hospital of Changsha, Changsha, Hunan China

**Keywords:** HMGCR, PCSK9, NPC1L1, Low-density lipoprotein cholesterol, Sarcopenia, Mendelian randomization

## Abstract

**Background:**

Lipid-lowering drugs are widely used among the elderly, with some studies suggesting links to muscle-related symptoms. However, the causality remains uncertain.

**Methods:**

Using the Mendelian randomization (MR) approach, we assessed the causal effects of genetically proxied reduced low-density lipoprotein cholesterol (LDL-C) through inhibitions of hydroxy-methyl-glutaryl-CoA reductase (HMGCR), proprotein convertase subtilisin/kexin type 9 (PCSK9), and Niemann-Pick C1-like 1 (NPC1L1) on sarcopenia-related traits, including low hand grip strength, appendicular lean mass, and usual walking pace. A meta-analysis was conducted to combine the causal estimates from different consortiums.

**Results:**

Using LDL-C pooled data predominantly from UK Biobank, genetically proxied inhibition of HMGCR was associated with higher appendicular lean mass (beta = 0.087, *P* = 7.56 × 10^− 5^) and slower walking pace (OR = 0.918, *P* = 6.06 × 10^− 9^). In contrast, inhibition of PCSK9 may reduce appendicular lean mass (beta = -0.050, *P* = 1.40 × 10^− 3^), while inhibition of NPC1L1 showed no causal impact on sarcopenia-related traits. These results were validated using LDL-C data from Global Lipids Genetics Consortium, indicating that HMGCR inhibition may increase appendicular lean mass (beta = 0.066, *P* = 2.17 × 10^− 3^) and decelerate walking pace (OR = 0.932, *P* = 1.43 × 10^− 6^), whereas PCSK9 inhibition could decrease appendicular lean mass (beta = -0.048, *P* = 1.69 × 10^− 6^). Meta-analysis further supported the robustness of these causal associations.

**Conclusions:**

Genetically proxied HMGCR inhibition may increase muscle mass but compromise muscle function, PCSK9 inhibition could result in reduced muscle mass, while NPC1L1 inhibition is not associated with sarcopenia-related traits and this class of drugs may serve as viable alternatives to sarcopenia individuals or those at an elevated risk.

**Supplementary Information:**

The online version contains supplementary material available at 10.1186/s40246-024-00643-3.

## Background

Lipid-lowering drugs have been extensively used in the management of atherosclerotic cardiovascular diseases among elderly individuals, with those targeting low-density lipoprotein cholesterol (LDL-C) being the most commonly prescribed in clinical practice [1]. Multiple drug classes exert distinct mechanisms to reduce LDL-C levels. First, statins inhibit hydroxy-methyl-glutaryl-CoA reductase (HMGCR), which are involved in the production of cholesterol in the cells [2]. Additionally, proprotein convertase subtilisin/kexin type 9 (PCSK9) normally binds to and degrades the receptors that remove LDL-C from the blood. By inhibiting this degradation process, PCSK9 inhibitors such as evolocumab and alirocumab augment the number of receptors and enhance the clearance of LDL-C from plasma [3]. Meanwhile, inhibitors targeting Niemann-Pick C1-like 1 (NPC1L1) protein can selectively inhibit small intestinal cholesterol transporters and thereby reduce the absorption of dietary cholesterol. By attenuating the influx of cholesterol into the bloodstream, ezetimibe and hybutimibe, representative drugs in this class, effectively lower LDL-C levels [4].

While the lipid-lowering effects of the drugs above have been well-documented, there is a growing concern regarding their potential long-term side effects. Observational researches have indicated that these commonly prescribed medications may lead to muscle pain, soreness, and/or weakness [5, 6]. Skeletal muscle-associated symptoms were reported by up to 20% of statin users [7]. A retrospective cohort study involving 477 participants revealed an incidence rate ranging from 32 to 36% for muscle-related adverse reactions associated with PCSK9 inhibitors [8]. Sarcopenia is a progressive and generalized skeletal muscle disorder characterized by accelerated loss of muscle strength, mass and function, leading to an increased risk of falls and hospitalization, particularly among the elderly population. A previous retrospective cohort study involving 586 sarcopenia patients reported an adverse association between statin use and recovery of muscle strength [9]. However, there exists inconsistency in the current clinical evidence, as another cross-sectional study including 136 heart failure patients found that statin use was associated with a reduced likelihood of developing sarcopenia [10].

Due to the heterogeneity in existing evidence and potential biases arising from inevitable reverse causation and confounding factors in observational data, it is challenging to determine causal associations in observational studies. Besides statins, the recent approval of PCSK9, and NPC1L1 inhibitors has led to a dearth of long-term adverse reaction data for risk of sarcopenia. Understanding these associations and further evaluating the causal impacts of lipid-lowering drugs on sarcopenia is crucial for guiding medication selection and personalized clinical care, particularly among the elderly population who require lipid-lowering drugs to manage metabolic or cardiovascular diseases and face an increased risk of sarcopenia with advancing age [11].

Mendelian randomization (MR) analysis, utilizing genetic variations associated with a specific exposure, presents a valuable alternative to supplement randomized clinical trials [12]. Genes that encode proteins capable of being modulated by drugs enable the implementation of drug target MR analysis. This is achieved by using genetic variants that mimic the effect of these target genes as instruments to infer causal associations between drug classes and diseases. Drug target MR analysis can reveal the long-term consequences of drug interventions and overcome confounding and reverse causation issues. This is attributed to the random allocation of genetic variants during conception, preceding the onset of diseases.

In this study, we aim to evaluate the causal impacts of the most commonly prescribed lipid-lowering drugs comprised of HMGCR, PCSK9, and NPC1L1 inhibitors on multiple sarcopenia-related traits via the MR approach and provide innovative recommendations for the clinical administration.

## Methods and materials

### Study design

To investigate the potentially causal associations between lipid-lowering drug classes and sarcopenia, we carried out a drug target MR study. In order to ensure the reliability of genetic variants as instrumental variables (IVs), three fundamental conditions in MR analysis must be satisfied. Firstly, the IVs should exhibit robust and consistent associations with the targeted exposure; secondly, these IVs must not be correlated with any potential confounders that could bias the exposure or outcome; and lastly, the IVs should exclusively influence the outcome through their direct impact on the exposure, while avoiding alternative pathways or external influences [13]. The data utilized in the present study were obtained from publicly accessible repositories. Ethical approval and informed consent were previously acquired in the original genome-wide association study (GWAS).

### Selection of IVs for lipid-lowering drug effect

**Fig. 1:**
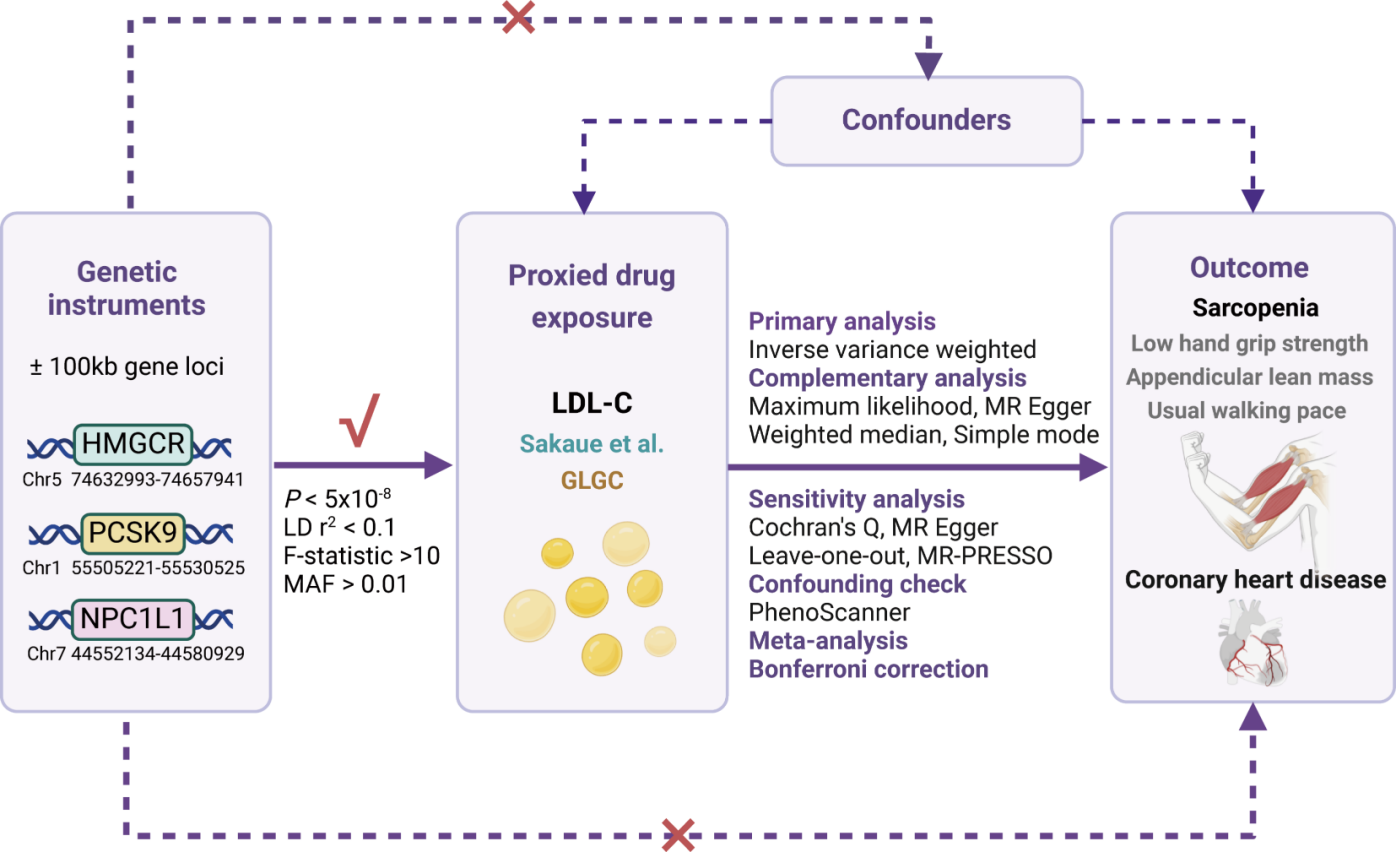
Study design overview. HMGCR, 3-hydroxy-3-methylglutaryl coenzyme A reductase; PCSK9, proprotein convertase subtilisin/kexin type 9; NPC1L1, Niemann-Pick C1-like 1; Chr, chromosome; LD, linkage disequilibrium; MAF, minor allele frequency; LDL-C, low-density lipoprotein cholesterol; GLGC, Global Lipids Genetics Consortium; MR-PRESSO, Mendelian randomization pleiotropy residual sum and outlier

The primary MR analysis leveraged summary-level dataset on LDL-C to proxy lipid-lowering drug exposure from a GWAS conducted by Sakaue et al., encompassing 416,487 individuals, primarily of European ancestry (343,621 Europeans and 72,866 East Asians). The dataset included 19,037,976 single nucleotide polymorphisms (SNPs) predominantly sourced from the UK Biobank [14]. To proxy the LDL-C lowering effects of HMGCR, PCSK9, and NCP1L1 inhibitions, we identified IVs based on the following criteria. These IVs were strategically selected SNPs located within a range of ± 100 kb from the genetic loci of HMGCR (Chromosome 5, 74,632,993–74,657,941), PCSK9 (Chromosome 1, 55,505,221–55,530,525), and NCP1L1 (Chromosome 7, 44,552,134–44,580,929) genes, exhibiting a statistically significant association with LDL-C levels (*P* < 5 × 10^− 8^) (Fig. 1 displays the overview of the study). In order to mitigate potential biases resulting from strong linkage disequilibrium (LD), we implemented an LD threshold of r^2^ < 0.1. The minimum minor allele frequency threshold of IVs was set as > 0.01. All SNPs designated as IVs in this study possessed a minimum F-statistic of 10 to counteract the potential influence of weak genetic instruments. Finally, a total of 9, 20, and 2 SNPs within the HMGCR, PCSK9, and NPC1L1 gene regions were selected, respectively. Additionally, for further validation, we utilized another summary-level GWAS dataset on LDL-C levels sourced from the Global Lipids Genetics Consortium (GLGC), encompassing 173,082 individuals of European ancestry with 2,437,752 SNPs [15]. We repeated the process outlined above to derive IVs to proxy the inhibitions of HMGCR, PCSK9, and NCP1L1 genes. A total of 2, 10, and 2 SNPs associated with LDL-C lowering within the HMGCR, PCSK9, and NPC1L1 gene regions were selected, respectively. Since both the primary and validation MR analyses included 2 SNPs to proxy NPC1L1 gene inhibition, we conducted an additional analysis with an LD threshold of r^2^ < 0.3 to enhance the robustness of our results. This led to the identification of 4 SNPs from the Sakaue et al. dataset and 3 SNPs from the GLGC dataset for subsequent causal estimations. Detailed information of IVs from LDL-C datasets is presented in the Supplementary Table S1-2.

### Source of outcomes

In our present MR analysis, according to the diagnostic recommendation of sarcopenia provided by the European Working Group on Sarcopenia in Older People (EWGSOP), we considered three sarcopenia-related traits as the outcomes, namely the low hand grip strength (60 years and older), appendicular lean mass, and usual walking pace. The summary-level GWAS dataset for low hand grip strength was sourced from the study conducted by Jones et al., encompassing data from 256,523 European participants with 9,336,415 SNPs across 22 cohorts [16]. The low hand grip strength was defined as grip strength < 30 kg for males and < 20 kg for females, which was measured using a hydraulic hand dynamometer in accordance with the diagnostic recommendation of EWGSOP. The GWAS dataset for appendicular lean mass was extracted from Pei et al.'s study, which included 450,243 European participants and 18,071,518 SNPs [17]. Appendicular lean mass representing the quantity of muscle, was defined as the summation of fat-free mass of arms and legs measured through bioelectrical impedance analysis. Appendicular lean mass raw values for all eligible participants were adjusted by appendicular fat mass, age, age squared, assessment center, and genotyping array. Appendicular lean mass is widely-recognized as a reliable metric for the approximation of muscle mass quantity in sarcopenia research [18]. According to the diagnostic guidelines proposed by EWGSOP, appendicular lean mass values below 20 kg for males and 15 kg for females are indicative of sarcopenia [19]. The GWAS dataset for usual walking pace, serving as an indicator of muscle function, was obtained from the European participants utilizing a linear model encompassing self-reported data of walking speed including slow (less than 3 miles per hour), steady (ranging from 3 to 4 miles per hour), and brisk (over 4 miles per hour) paces. The cut-off speed lower than 0.8 m per second (equivalent to 1.79 miles per hour) is advised by EWGSOP as an indicator of severe sarcopenia [19]. This dataset consisted of genetic information from 459,915 individuals and comprised a total of 9,851,867 SNPs.

Moreover, we additionally used a GWAS dataset on coronary heart disease (CHD) [20] from the European ancestry as the positive control, demonstrating the effectiveness of proxied LDL-C lowering through drug target genes inhibitions in MR analysis. A summary of the GWAS datasets utilized in the study is presented in Supplementary Table S3.

### Data analysis and statistics

After harmonizing the selected IVs with the datasets of sarcopenia-related traits and excepting palindromic SNPs with intermediate allele frequencies, a drug target MR analysis was performed. We primarily employed the inverse-variance weighted (IVW) method, which has been widely recognized as the most powerful approach in MR analysis [21], to investigate the potential causative links between proxied LDL-C lowering drug exposure and sarcopenia-related traits. When there was heterogeneity among IVs, the IVW-multiplicative random effects model may be used. Otherwise, IVW-fixed effects model would be applied. Additionally, we incorporated several complementary methods, including MR Egger, weighted median, maximum likelihood, and simple mode. MR Egger is suitable for causal estimation when pleiotropy among IVs is present. Weighted median infers the causality by considering the weights of SNP-specific estimates, ensuring robustness in case of limited number of valid IVs. Maximum likelihood estimation addresses bias stemming from sample overlap, while simple mode offers unweighted mode of the empirical density function of causal estimates [22, 23]. All statistical computations were conducted using the “TwoSampleMR” (Version 0.5.7) and “MRInstruments” (Version 0.3.2) packages within RStudio (Version 2023.06.0). For each standard deviation decrease in LDL-C levels, the odds ratio (OR) indicates the risk of categorical outcomes (low hand grip strength, usual walking pace), while the beta coefficient quantifies the number of standard deviation changes of the continuous outcome (appendicular lean mass in kilogram). After applying the Bonferroni multiple testing correction for genetically proxied drug exposure and sarcopenia-related traits, we established a significance threshold for a *P*-value below 5.56 × 10^− 3^ (*P* = 0.05/9) to indicate significant evidence of a causal association.

### Sensitivity analysis

To gauge the degree of heterogeneity within the IVs, we employed Cochran’s Q test. MR Egger regression was used to test the possibility of horizontal pleiotropy among the IVs [24]. Heterogeneity and pleiotropy were taken into account with a *P*-value below 0.05. The online tool PhenoScanner was used to thoroughly examine all IVs, ensuring that all selected IVs for MR analysis were not associated with confounding factors (such as smoking, alcohol use, physical activity, body weight, body mass index, etc.). No SNP was removed in this step. To minimize the potential influence of each SNP on the overall results, the MR pleiotropy residual sum and outlier (MR-PRESSO) as well as leave-one-out analysis was utilized to identify potential outliers among the IVs [24, 25]. In instances where an SNP outlier was detected via MR-PRESSO or leave-one-out, the outlier should be excluded. The MR analysis may be subsequently reiterated to derive the ultimate robust outcomes. The detailed information of excluded SNPs is presented in Supplementary Table S4.

As the outcome GWAS datasets of appendicular lean mass and usual walking pace were predominantly sourced from participants of the UK Biobank, we took into account the influence of sample overlap when conducting MR analysis using LDL-C from the GWAS of Sakaue et al. to proxy lipid-lowering drug exposure. To ensure the reliability of the results, we utilized the “mrSampleOverlap” (Version 0.1.1) package within RStudio to estimate the bias caused by sample overlap and the probability of Type 1 error [26].

### Meta-analysis

Additionally, to provide a more comprehensive interpretation of the above findings. we conducted a meta-analysis by merging the IVW causal estimates using LDL-C from Sakaue et al., as well as GLGC to proxy the lipid-lowering drug exposure of HMGCR, PCSK9, and NCP1L1 inhibitions on sarcopenia-related traits through the Review Manager software (Version 5.3).

## Results

### Positive control analysis

In the IVW estimates, using LDL-C from the GWAS of Sakaue et al., genetically proxied inhibition of HMGCR (IVW, OR [95%CI] = 0.620 [0.496–0.775], *P* = 2.77 × 10^− 5^), PCSK9 (IVW, OR [95%CI] = 0.449 [0.379–0.531], *P* = 8.72 × 10^− 21^), and NPC1L1 (IVW, OR [95%CI] = 0.456 [0.264–0.788], *P* = 4.91 × 10^− 3^) demonstrated significant causal associations with the reduced risk of CHD as expected. Consistent findings were also demonstrated by complementary MR methods. The MR findings using LDL-C from the GLGC to proxy lipid-lowering drug exposure further substantiated these causal associations. This indicated that our model of lipid-lowering drug target MR analysis was successfully established. The detailed results of the significant causal associations between lipid-lowering drug exposure and CHD are presented in Table 1.

**Table 1:** The causal estimations of genetically proxied inhibitions of HMGCR, PCSK9, and NPC1L1 on coronary heart disease

Dataset	Drug target	Method	nSNPs	OR (95%CI)	*P*-value
Sakaue et al.	HMGCR	Inverse variance weighted (fixed effects)	9	0.620 (0.496 to 0.775)	**2.77E-05**
		Weighted median	9	0.655 (0.495 to 0.867)	**3.05E-03**
		Maximum likelihood	9	0.619 (0.494 to 0.775)	**3.03E-05**
		MR Egger	9	0.373 (0.181 to 0.769)	**3.19E-02**
		Simple mode	9	0.896 (0.553 to 1.452)	6.68E-01
	PCSK9	Inverse variance weighted (fixed effects)	20	0.449 (0.379 to 0.531)	**8.72E-21**
		Weighted median	20	0.469 (0.372 to 0.592)	**1.57E-10**
		Maximum likelihood	20	0.458 (0.386 to 0.543)	**2.37E-19**
		MR Egger	20	0.442 (0.310 to 0.630)	**2.65E-04**
		Simple mode	20	0.430 (0.299 to 0.618)	**2.12E-04**
	NPC1L1	Inverse variance weighted (fixed effects)	2	0.456 (0.264 to 0.788)	**4.91E-03**
		Maximum likelihood	2	0.455 (0.260 to 0.795)	**5.68E-03**
GLGC	HMGCR	Inverse variance weighted (fixed effects)	2	0.688 (0.557 to 0.851)	**5.48E-04**
		Maximum likelihood	2	0.685 (0.551 to 0.852)	**6.56E-04**
	PCSK9	Inverse variance weighted (fixed effects)	10	0.601 (0.532 to 0.680)	**4.79E-16**
		Weighted median	10	0.597 (0.501 to 0.710)	**6.07E-09**
		Maximum likelihood	10	0.599 (0.528 to 0.680)	**1.45E-15**
		MR Egger	10	0.550 (0.399 to 0.756)	**6.23E-03**
		Simple mode	10	0.557 (0.410 to 0.758)	**4.78E-03**
	NPC1L1	Inverse variance weighted (fixed effects)	2	0.590 (0.397 to 0.876)	**8.98E-03**
		Maximum likelihood	2	0.589 (0.392 to 0.883)	**1.05E-02**

Bold font indicates the statistical significance of a causal effect. nSNPs, number of single-nucleotide polymorphisms; OR, odd ratio; CI, confidence interval; GLGC, Global Lipids Genetics Consortium; HMGCR, 3-hydroxy-3-methylglutaryl coenzyme A reductase; PCSK9, proprotein convertase subtilisin/kexin type 9; NPC1L1, Niemann-Pick C1-Like 1

### Causal estimates of lipid-lowering drugs on low hand grip strength

**Fig. 2:**
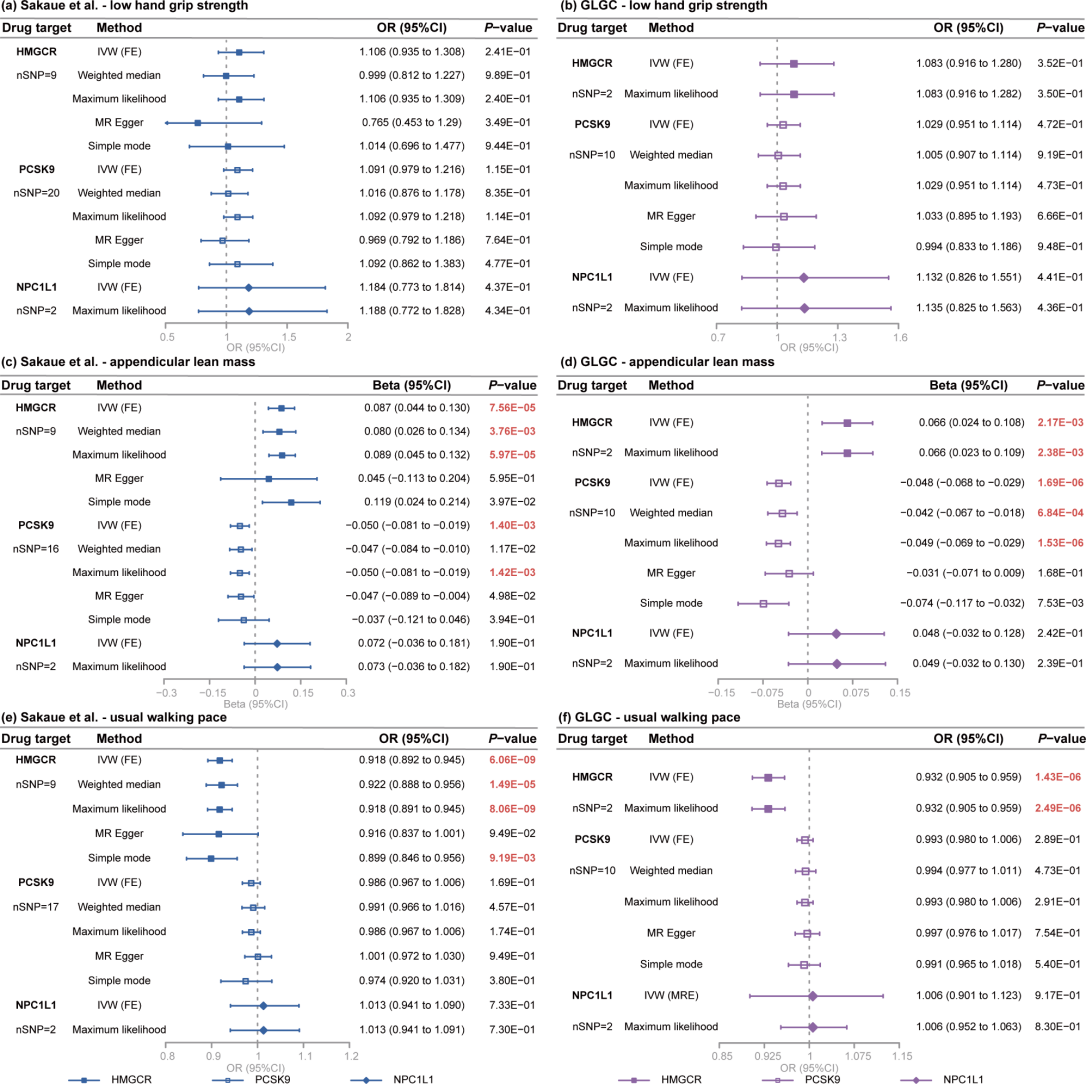
Forest plots illustrate the causal estimates from the MR investigation employing LDL-C decrease as a proxy for lipid-lowering drug effects. The findings indicate that genetically proxied inhibitions of HMGCR, PCSK9, and NPC1L1 may not causally influence low hand grip strength based on LDL-C datasets from Sakaue et al. (**a**), as well as GLGC (**b**). Genetically proxied inhibition of HMGCR correlates with an increase in appendicular lean mass, while inhibition of PCSK9 associates with a decrease in appendicular lean mass, according to LDL-C datasets from Sakaue et al. (**c**), and GLGC (**d**). Additionally, genetically proxied inhibition of HMGCR is linked to decelerating walking pace based on LDL-C datasets from Sakaue et al. (**e**), and GLGC (**f**). Genetically proxied inhibition of NPC1L1 has no causal impact on sarcopenia-related traits. *P*-values in bold and red to indicate the results with statistical significance. MR, Mendelian randomization; OR, odds ratio; CI, confidence interval; IVW, inverse variance weighted; FE, fixed effects; MRE, multiplicative random effects; HMGCR, 3-hydroxy-3-methylglutaryl coenzyme A reductase; PCSK9, proprotein convertase subtilisin/kexin type 9; NPC1L1, Niemann-Pick C1-like 1; LDL-C, low-density lipoprotein cholesterol; GLGC, Global Lipids Genetics Consortium

In the MR analysis, using LDL-C from Sakaue et al. (Fig. 2a), as well as from the GLGC (Fig. 2b) to proxy lipid-lowering drug exposure, we observed no causal association of genetically proxied inhibitions of HMGCR, PCSK9, and NPC1L1 with the risk of low hand grip strength.

### Causal estimates of lipid-lowering drugs on appendicular lean mass

**Fig. 3:**
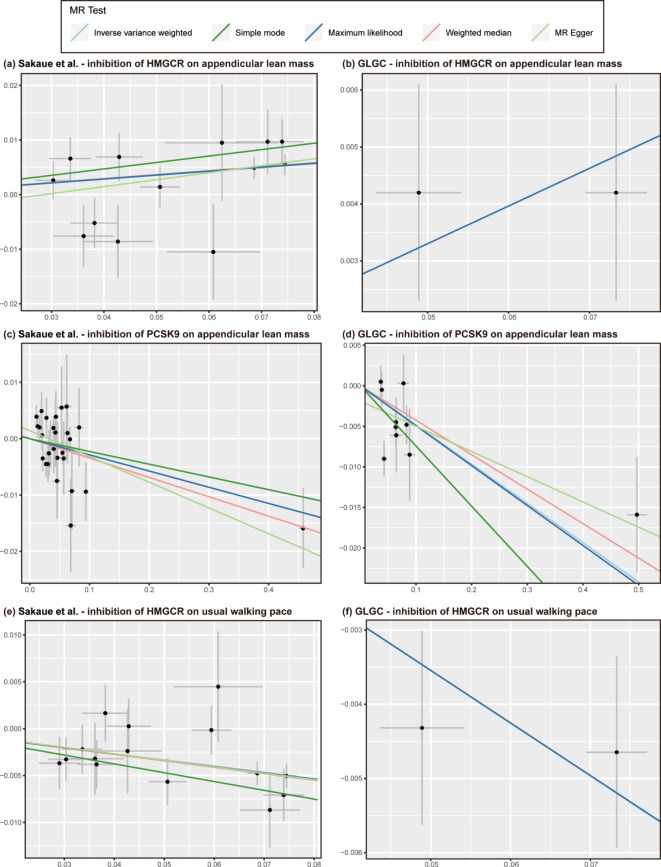
Scatter plots depict the SNP effect of genetically proxied LDL-C decrease (x-axis) on the risk of sarcopenia related-traits (y-axis), which indicate that inhibition of HMGCR is related to an increase in appendicular lean mass using LDL-C datasets from Sakaue et al. (**a**) and GLGC (**b**). In contrast, inhibition of PCSK9 is significantly associated with a decrease in appendicular lean mass using LDL-C datasets from Sakaue et al. (**c**) and GLGC (**d**). Inhibition of HMGCR is associated with a decelerated walking pace using LDL-C datasets from Sakaue et al. (**e**) and GLGC (**f**). MR, Mendelian randomization; HMGCR, 3-hydroxy-3-methylglutaryl coenzyme A reductase; PCSK9, proprotein convertase subtilisin/kexin type 9; NPC1L1, Niemann-Pick C1-like 1; LDL-C, low-density lipoprotein cholesterol; GLGC, Global Lipids Genetics Consortium

Using LDL-C from Sakaue et al. to proxy lipid-lowering drug exposure, inhibition of HMGCR (IVW, beta [95%CI] = 0.087 [0.044 to 0.130], *P* = 7.56 × 10^− 5^) was demonstrated to significantly increase the appendicular lean mass. This conclusion was confirmed by complementary MR methods including weighted median (beta [95%CI] = 0.080 [0.026 to 0.134], *P* = 3.76 × 10^− 3^) and maximum likelihood (beta [95%CI] = 0.089 [0.045 to 0.132], *P* = 5.97 × 10^− 5^) (Figs. 2c and 3a). Meanwhile, genetically proxied inhibition of PCSK9 (IVW, beta [95%CI] =-0.050 [-0.081 to-0.019], *P* = 1.40 × 10^− 3^) was found to significantly reduce the appendicular lean mass, confirmed by maximum likelihood method (beta [95%CI] = -0.050 [-0.081 to -0.019], *P* = 1.42 × 10^− 3^) (Figs. 2c and 3c). Genetically proxied inhibition of NPC1L1 did not show any causal association with appendicular lean mass (Fig. 2c).

The alike results were obtained in the analysis using LDL-C from the GLGC to proxy lipid-lowering drug exposure, which suggested that inhibition of HMGCR would increase the appendicular lean mass (IVW, beta [95%CI] = 0.066 [0.024 to 0.108], *P* = 2.17 × 10^− 3^), confirmed by maximum likelihood method (beta [95%CI] = 0.066 [0.023 to 0.109], *P* = 2.38 × 10^− 3^) (Figs. 2d and 3b). In contrast, inhibition of PCSK9 may significantly decrease the appendicular lean mass (IVW, beta [95%CI] = -0.048 [-0.068 to -0.029], *P* = 1.69 × 10^− 6^), confirmed by complementary MR methods including weighted median (beta [95%CI] = -0.042 [-0.067 to -0.018], *P* = 6.84 × 10^− 4^) and maximum likelihood (beta [95%CI] = -0.049 [-0.069 to -0.029], *P* = 1.53 × 10^− 6^) (Figs. 2d and 3d).

### Causal estimates of lipid-lowering drugs on usual walking pace

Using LDL-C from the Sakaue et al. to proxy lipid-lowering drug exposure, inhibition of HMGCR (IVW, OR [95%CI] = 0.918 [0.892 to 0.945], *P* = 6.06 × 10^− 9^) was observed to possess a significantly adverse causal effect on usual walking pace. This conclusion was also confirmed by complementary MR methods including weighted median (OR [95%CI] = 0.922 [0.888 to 0.956], *P* = 1.49 × 10^− 5^) and maximum likelihood (OR [95%CI] = 0.918 [0.891 to 0.945], *P* = 8.06 × 10^− 9^) (Figs. 2e and 3e). Genetically proxied inhibition of PCSK9 and NPC1L1 may not be causally associated with walking pace (Fig. 2e).

Using LDL-C from the GLGC to proxy lipid-lowering drug exposure, we also confirmed that inhibition of HMGCR may have a significantly adverse causal impact on usual walking pace (IVW, OR [95%CI] = 0.932 [0.905 to 0.959], *P* = 1.43 × 10^− 6^). Maximum likelihood also supported such a finding (OR [95%CI] = 0.932 [0.905 to 0.959], *P* = 2.49 × 10^− 6^) (Figs. 2f and 3f).

For the NPC1L1 inhibition, additional analysis using an LD threshold of r^2^ < 0.3 to increase the number of SNPs as instruments revealed a potential causal association with a lower risk of CHD (Supplementary Table S5), and no causal association with any of the three sarcopenia-related traits (Supplementary Table S6). These results are highly consistent with our primary findings. Additionally, the scatter plots for the causal estimates of inhibitions of HMGCR, PCSK9, and NPC1L with sarcopenia-related traits, which did not show significant correlations, are presented in Supplementary Figures S1-S6. The forest plots for all the causal estimates of inhibitions of HMGCR, PCSK9, and NPC1L with sarcopenia-related traits are presented in Supplementary Figures S7-S12.

### Sensitivity analysis

To test the robustness of the MR study, we used Cochran’s Q and MR Egger test to assess the heterogeneity and horizontal pleiotropy. We detected heterogeneity (*P* = 0.041) when investigating the causality between the inhibition of NPC1L1 and usual walking pace in the analysis using LDL-C from the GLGC to proxy the drug effect. Notably, the IVW-multiplicative random effects method we adopted could eliminate the bias caused by heterogeneity among the IVs [27]. All the other results revealed no evidence of heterogeneity or horizontal pleiotropy among IVs. For our findings, leave-one-out analysis has provided a strong support for robustness with no SNP outlier detected in the MR analysis using LDL-C dataset from Sakaue et al. (Supplementary Figure S13-S15), as well as GLGC (Supplementary Figure S16). The detailed results for sensitivity analysis are presented in Supplementary Table S7 and S8.

The evaluation regarding the influence of sample overlap between datasets of LDL-C (Sakaue et al.) and appendicular lean mass, as well as usual walking pace, demonstrated that with increasing rates of sample overlap, there may be a corresponding increase in bias and the likelihood of Type I error (Supplementary Figure S17 for appendicular lean mass; Supplementary Figure S18 for usual walking pace). The estimated maximum sample overlap proportions between the LDL-C and appendicular lean mass, usual walking pace datasets were 76.3% and 74.7%. For the maximum overlapping ratios, the bias and Type I error rate in causal estimations were below 2.75 × 10^− 3^ and 5.74 × 10^− 2^ for appendicular lean mass, (Supplementary Table S9), and below 2.66 × 10^− 3^ and 5.69 × 10^− 2^ for usual walking pace, respectively (Supplementary Table S10). These findings suggest that the impact of sample overlap on the results of MR analysis may be minimal and acceptable.

### Result of meta-analysis

**Fig. 4:**
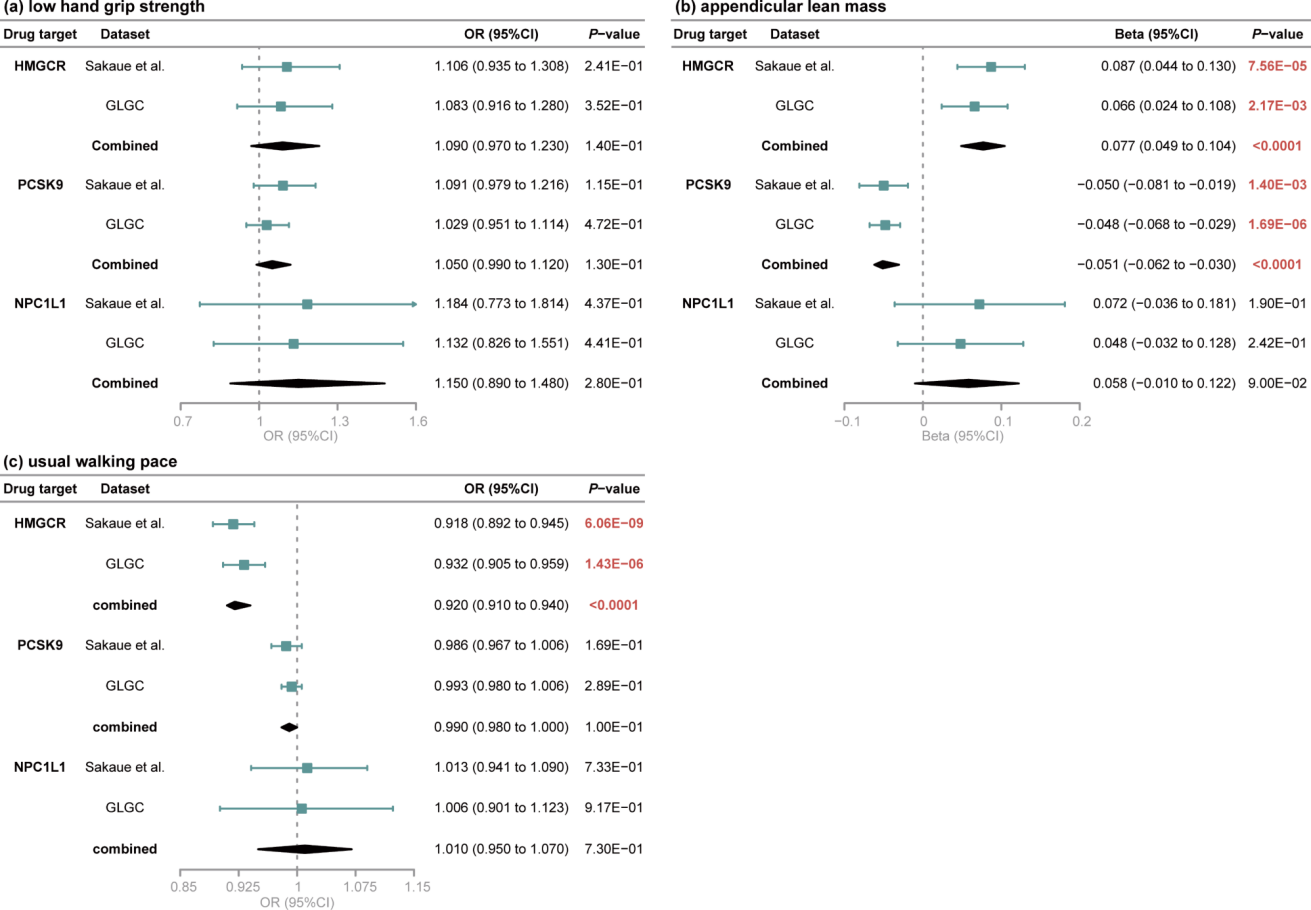
A meta-analysis was conducted by merging the inverse-variance weighted causal estimates obtained from MR analysis using LDL-C datasets from Sakaue et al., as well as GLGC. The plots illustrate the combined estimates for inhibitions of HMGCR, PCSK9, and NPC1L on sarcopenia-related traits. Genetically proxied inhibitions of HMGCR, PCSK9, and NPC1L1 have no causal impact on risk of low hand grip strength (**a**). Genetically predicted inhibition of HMGCR is related to an increase in appendicular lean mass, while inhibition of PCSK9 is associated with a decrease in appendicular lean mass (**b**). Genetically proxied inhibition of HMGCR is also associated with a decelerated walking pace (**c**). *P*-values in bold and red to indicate the results with statistical significance. OR, odds ratio; CI, confidence interval; HMGCR, 3-hydroxy-3-methylglutaryl coenzyme A reductase; PCSK9, proprotein convertase subtilisin/kexin type 9; NPC1L1, Niemann-Pick C1-like 1; LDL-C, low-density lipoprotein cholesterol; GLGC; Global Lipids Genetics Consortium

After conducting a meta-analysis to combine the IVW estimates from two LDL-C related GWAS datasets obtained from Sakaue et al., as well as GLGC, we observed a significant causal association between genetically proxied inhibition of HMGCR and an increase in appendicular lean mass (beta [95%CI] = 0.077 [0.049 to 0.104], *P* < 1.00 × 10^− 4^). Genetically proxied inhibition of HMGCR also exhibited a significantly adverse causal impact on usual walking pace (OR [95%CI] = 0.920 [0.910 to 0.940], *P* < 1.00 × 10^− 4^). Furthermore, genetically proxied inhibition of PCSK9 was causally associated with a decrease in appendicular lean mass (beta [95%CI] = -0.051 [-0.062 to -0. 030], *P* < 1.00 × 10^− 4^) (Fig. 4). The merged estimates in meta-analysis were highly consistent with the results from MR analysis.

## Discussion

In this study, we conducted comprehensive drug target MR analysis to evaluate the causal effects of the most commonly prescribed lipid-lowering drug classes including HMGCR, PCSK9, and NPC1L1 inhibitors on sarcopenia risk. To further validate our findings, a meta-analysis was performed to combine the causal estimates following the MR analysis from different consortiums. According to the findings, genetically proxied HMGCR inhibition demonstrated significant causal associations with an increase in appendicular lean mass but decelerated walking pace. Meanwhile, genetically proxied PCSK9 inhibition exhibited a robust causal effect on the reduction of appendicular lean mass. Genetically proxied inhibition of NPC1L1 demonstrated no causal association with all the three sarcopenia-related traits.

Statin medications, the most widely used lipid-lowering drug [28], target the enzyme HMGCR, vital in cholesterol synthesis. By inhibiting HMGCR, statins reduce cholesterol production in the liver, effectively lowering LDL-C levels. Additionally, statins would possess secondary benefits, such as anti-inflammatory properties and plaque stabilization. Commonly prescribed statins, including atorvastatin and simvastatin, are vital in treating individuals with elevated LDL-C levels or a history of cardiovascular disease. On the other hand, statins have been well-documented for its muscle-related adverse reactions, like myopathies, rhabdomyolysis, etc. [29]. 5–20% of statin users complained about mild to moderate skeletal muscle-associated symptoms [7]. Despite of high prevalence of such adverse effect, the impact of stains on muscle function remained inconclusive. Our research indicated that long-term administration of statins could compromise muscle function manifested by decelerated walking pace. A previous work by Parker et al. [30]. found that statin users with self-reported muscle symptoms such as hip flexor, quadriceps, hamstring and/or calf aches (*n* = 18) experienced a decline in 5 out of 14 performance measures, but they also observed a similar deterioration in four performance measures among placebo individuals with muscle symptoms. Another clinical research comprised of 92 statin users reported modest improvements in muscle function concurrent with a decrease in subjective symptom intensity after statin withdrawal [31]. On the other hand, there is indeed inconsistency in existing evidence, with some clinical research reporting statins have no impact on muscle strength [32, 33] or muscle mass [33]. The detailed mechanisms leading to muscle symptoms following statin use were not fully clarified. Statins have been proven to be associated with mitochondrial dysfunction, which may be the most likely cause of statin-associated muscle symptoms [34]. Mitochondrial dysfunction by statins may be responsible for the activated protein kinase, which could be associated with impaired activation of rapamycin complex 1, leading to accelerated skeletal muscle protein degradation, impaired protein synthesis, and stimulation of apoptosis [29].

PCSK9 inhibitors represent a newer class of medications that target the PCSK9 protein, regulating LDL-C receptor levels in liver cells. By inhibiting PCSK9, medications like evolocumab and alirocumab increase the availability of LDL receptors, enhancing LDL-C clearance. Typically administered as adjunctive therapy, PCSK9 inhibitors are particularly useful in individuals with familial hypercholesterolemia or those requiring additional LDL-C lowering despite maximum statin therapy. However, our MR findings indicated that genetically proxied inhibition of PCSK9 may actually increase the risk of sarcopenia, characterized by significantly reducing the muscle mass. A retrospective cohort by Donald et al. comprised of 477 individuals using PCSK9 inhibitor found an incidence rate of 36% for muscle-related adverse reactions like myalgia [8]. Another retrospective cohort study comprised of 137 participants reported that 24 patients (17.5%) developed muscle-related adverse reactions such as myalgia, myopathies, etc. [35]. The most commonly used PCSK9 inhibitors, evolocumab and alirocumab, were found to be associated with musculoskeletal and connective tissue disorders (including back pain, myalgia, pain in extremity, arthralgia, muscle spasms), in a recent retrospective study based on the Food and Drug Administration adverse event reporting system database [36].

Overall, these existing researches are retrospective, mostly of single-center and limited sample size. Considering the fact that PCSK9 inhibitors have just been approved for clinical use for less than a decade [37], it may be challenging to determine their long-term effects and adverse reactions at this time. Our results and existing evidence call for further caution in muscle-related adverse reactions of PCSK9 inhibitors that may increase the risk of sarcopenia.

Ezetimibe, another key medication, acts on the NPC1L1 protein in the small intestine, inhibiting dietary cholesterol absorption. Often used alongside statins, ezetimibe further reduces LDL-C levels, especially in individuals who are intolerant to high statin doses or who require an alternative therapy. Administered orally, ezetimibe highlights the importance of multifaceted approaches in managing dyslipidemia and cardiovascular risk. The potential effects of NPC1L1 inhibitors on muscle strength, quantity, and function remain largely uncertain. One previous randomized clinical trial comprised of 491 participants [38] reported 28.8% of ezetimibe-treated patients exhibited muscle symptoms, including myalgia, muscle spasms. Another previous study conducted on a mouse model of muscular dystrophy suggested that ezetimibe may exhibit a protective effect against muscle wasting, potentially attributed to its ability to mitigate cholesterol-induced damage in weakened myofibers, suppress inflammatory cytokine signaling, and regulate macrophage recruitment [39]. On the other hand, another study of 827 participants reported that musculoskeletal symptoms in only 3% of the Ezetimibe arm versus 4% of the placebo arm during 12 weeks of treatment [40], indicating that there may be no muscle-related adverse effects regarding NPC1L1 inhibitors. Considering the extensively documented muscle-related adverse effects associated with both HMGCR and PCSK9 inhibitors based on previous observational studies and our MR findings, we proposed that the NPC1L1 inhibitors, such as ezetimibe, could potentially serve as relatively viable alternative lipid-lowering drugs for individuals with sarcopenia or those at an elevated risk.

This study represents the pioneering effort to comprehensively analyze the causal effects of commonly prescribed lipid-lowering drug classes on sarcopenia-related traits. However, it is important to acknowledge some limitations. Firstly, our analysis was based solely on GWAS of individuals with European ancestry, which may restrict the generalizability of our findings to other populations. Secondly, drug target MR analysis could only simulate the long-term genetically predicted effects of drug classes on disease, while the short-term effects may be inestimable. Thirdly, factors such as drug dosage, interindividual variability in drug metabolism, and drug-binding affinity might affect both toxicity and efficacy outcomes, which could not be further analyzed in our present study. Additionally, other covariates such as height, sex, baseline appendicular lean mass, and baseline walking pace must be taken into account in diagnosing and evaluating sarcopenia in clinical settings. Therefore, MR analysis alone may not fully capture the overall impact of drug exposure. While our findings offered valuable insights, large-scale and well-designed cohort studies in real-world settings are still imperative to examine the causal effects of long-term use of HMGCR, PCKS9, and NPC1L1 inhibitors on sarcopenia.

## Conclusion

In conclusion, this MR study provides evidence supporting the causal roles of certain lipid-lowering drugs in the development of sarcopenia from a genetic standpoint. Extra caution should be taken when administering these drugs to elderly individuals. It highlights that long-term use of HMGCR inhibitors may increase muscle mass but compromise muscle function, and PCSK9 inhibitors could result in reduction of muscle mass, while NPC1L1 inhibitors are not associated with sarcopenia-related traits and may serve as viable alternatives to individuals with sarcopenia or those at an elevated risk.

### Electronic supplementary material

Below is the link to the electronic supplementary material.

Supplementary Material 1

Supplementary Material 2

## Data Availability

No datasets were generated or analysed during the current study.

## Abbreviations

LDL-C: Low Density Lipoprotein Cholesterol
HMGCR: Hydroxy Methyl-Glutaryl-Coa Reductase
PCSK9: Proprotein Convertase Subtilisin/Kexin type 9
NPC1L1: Niemann-Pick C1-Like 1
MR: Mendelian Randomization
IVs: Instrumental Variables
GWAS: Genome-Wide Association Study
SNPs: Single Nucleotide Polymorphisms
LD: Linkage Disequilibrium
GLGC: Global Lipids Genetics Consortium
EWGSOP: European Working Group on Sarcopenia in Older People
CHD: Coronary Heart Disease
IVW: Inverse-Variance Weighted
OR: Odds Ratio
MR-PRESSO: Mendelian Randomization Pleiotropy Residual Sum and Outlier
