# Supplementary material for: Association of lipid-lowering drugs with risk of sarcopenia: a drug target mendelian randomization study and meta-analysis
Source: Hum Genomics. 2024 Jul 3;18:76. doi: 10.1186/s40246-024-00643-3 (PMC11223278; doi:10.1186/s40246-024-00643-3)
Supplement: Supplementary file 1 — Supplementary Material 1 [file 40246_2024_643_MOESM1_ESM.pdf]

(A) inhibition of HMGCR on low hand grip strength

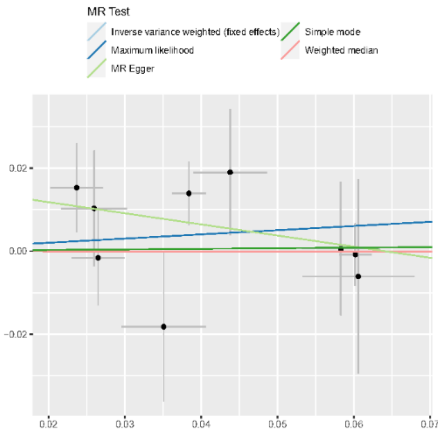

(B) inhibition of PCSK9 on low hand grip strength

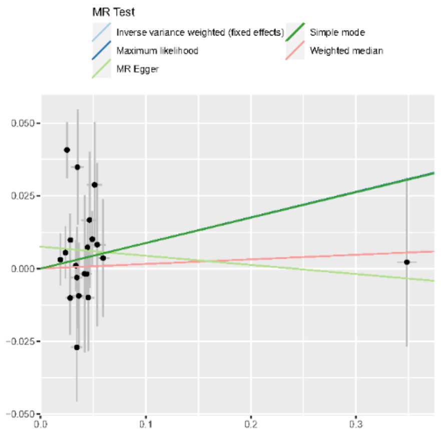

(C) inhibition of NPC1L1 on low hand grip strength

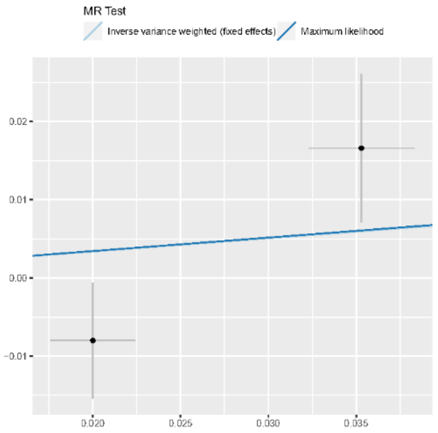

(D) inhibition of NPC1L1 on low hand grip strength*

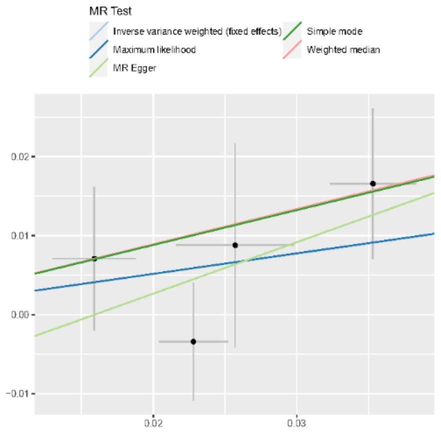

Supplementary Figure S1. Scatter plots depict the SNP effect associated with inhibitions of HMGCR, PCSK9, and NPC1L1 (x-axis) on low hand grip strength (y-axis) using LDL-C from the GWAS of Sakaue et al. to proxy drug exposure. *Linkage disequilibrium (LD) threshold of $r^2<0.3$ in the selection of instrumental variables.

(A) inhibition of NPC1L1 on appendicular lean mass

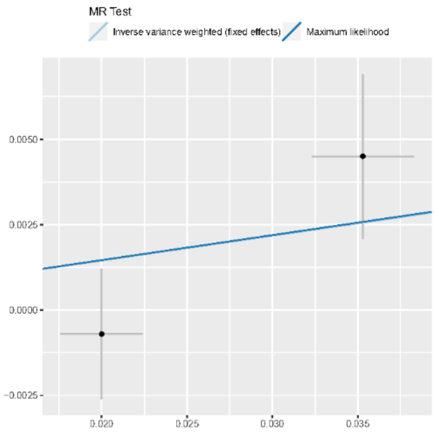

(B) inhibition of NPC1L1 on appendicular lean mass*

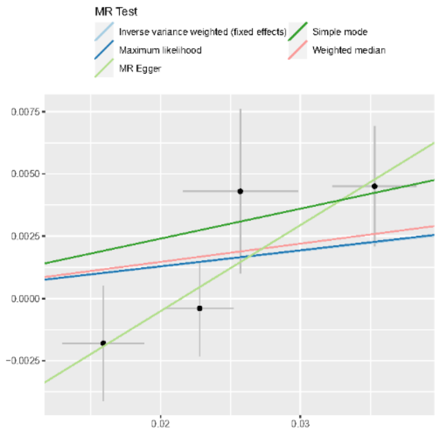

Supplementary Figure S2. Scatter plot depicts the SNP effect associated with inhibition of NPC1L1 (x-axis) on appendicular lean mass (y-axis) using LDL-C from the GWAS of Sakaue et al. to proxy drug exposure. *Linkage disequilibrium (LD) threshold of $r^2<0.3$ in the selection of instrumental variables.

(A) inhibition of PCSK9 on usual walking pace

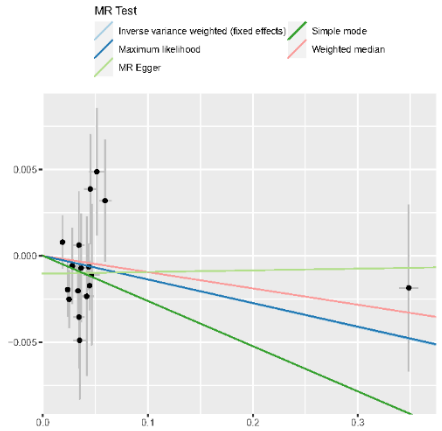

(B) inhibition of NPC1L1 on usual walking pace

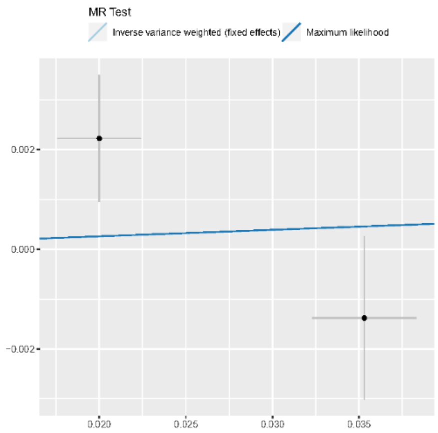

(C) inhibition of NPC1L1 on usual walking pace*

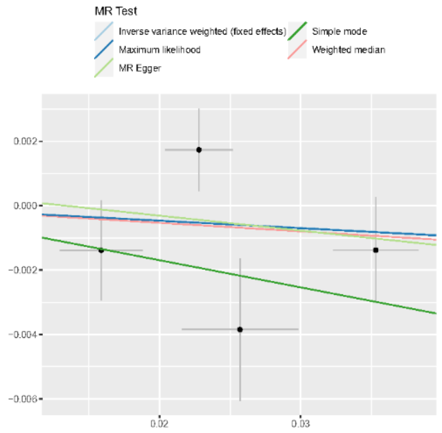

Supplementary Figure S3. Scatter plots depict the SNP effect associated with inhibitions of PCSK9 and NPC1L1 (x-axis) on usual walking pace (y-axis) using LDL-C from the GWAS of Sakaue et al. to proxy drug exposure. *Linkage disequilibrium (LD) threshold of $r^2<0.3$ in the selection of instrumental variables.

(A) inhibition of HMGCR on low hand grip strength

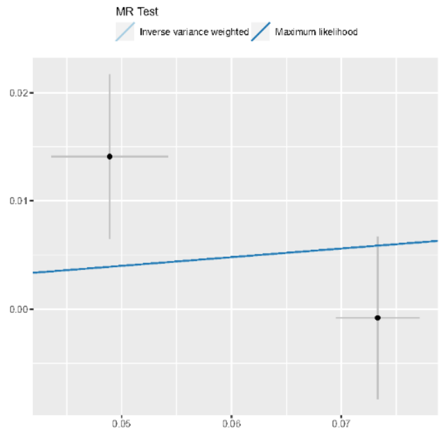

(B) inhibition of PCSK9 on low hand grip strength

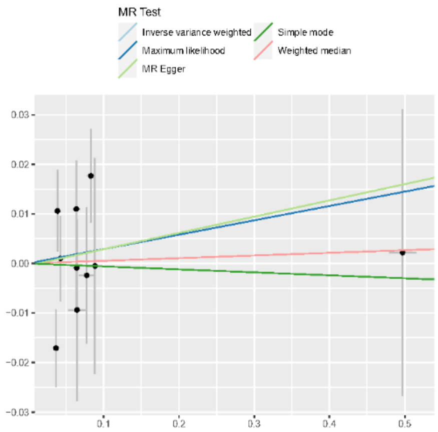

(C) inhibition of NPC1L1 on low hand grip strength

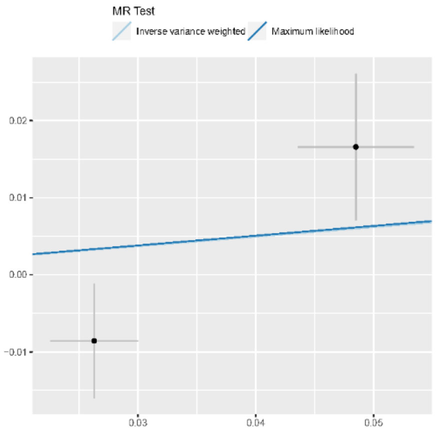

(D) inhibition of NPC1L1 on low hand grip strength*

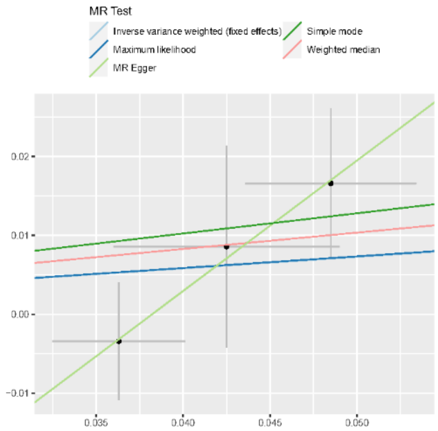

Supplementary Figure S4. Scatter plots depict the SNP effect associated with inhibitions of HMGCR, PCSK9, and NPC1L1 (x-axis) on low hand grip strength (y-axis) using LDL-C from the GLGC to proxy drug exposure. *Linkage disequilibrium (LD) threshold of r^2<0.3 in the selection of instrumental variables.

(A) inhibition of NPC1L1 on appendicular lean mass

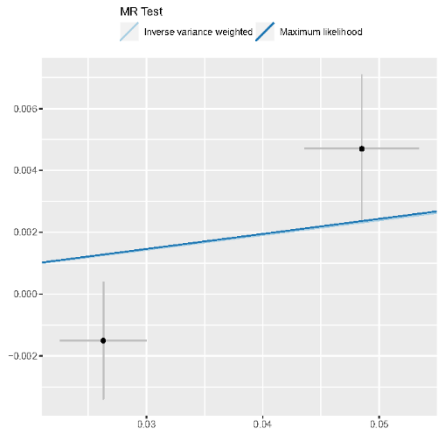

(B) inhibition of NPC1L1 on appendicular lean mass*

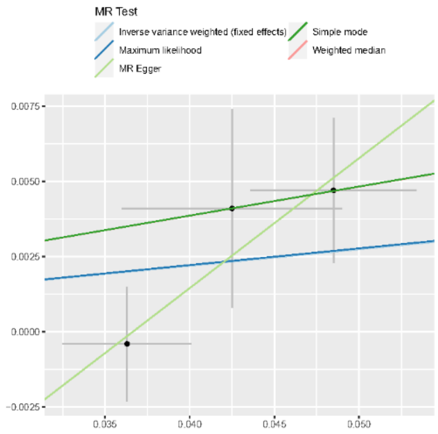

Supplementary Figure S5. Scatter plot depicts the SNP effect associated with inhibition of NPC1L1 (x-axis) on appendicular lean mass (y-axis) using LDL-C from the GLGC to proxy drug exposure. *Linkage disequilibrium (LD) threshold of $r^2<0.3$ in the selection of instrumental variables.

(A) inhibition of PCSK9 on usual walking pace

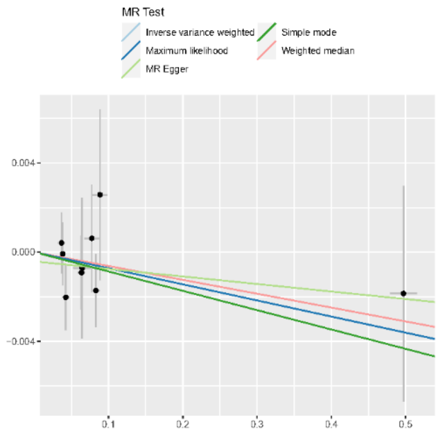

(B) inhibition of NPC1L1 on usual walking pace

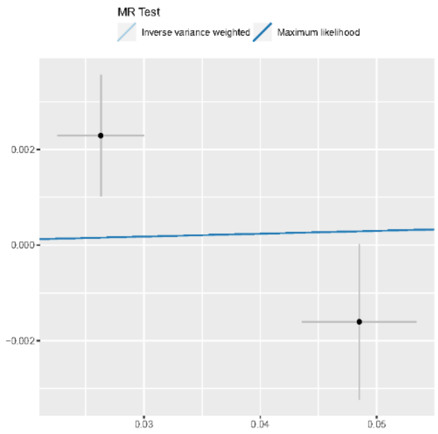

(C) inhibition of NPC1L1 on usual walking pace*

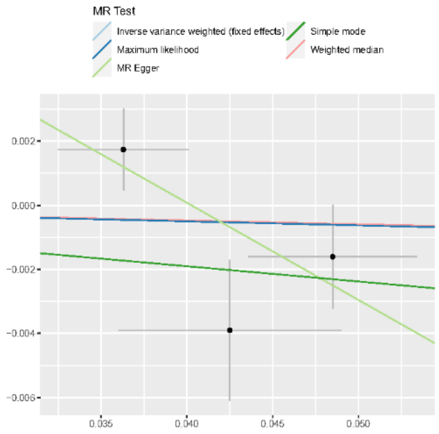

Supplementary Figure S6. Scatter plots depict the SNP effect associated with inhibitions of PCSK9 and NPC1L1 (x-axis) on usual walking pace (y-axis) using LDL-C from the GLGC to proxy drug exposure. *Linkage disequilibrium (LD) threshold of r^2<0.3 in the selection of instrumental variables.

(A) inhibition of HMGCR on low hand grip strength

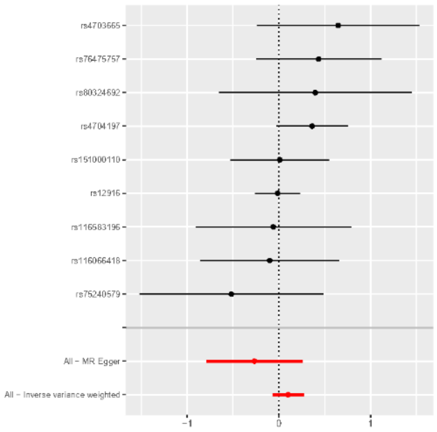

(B) inhibition of PCSK9 on low hand grip strength

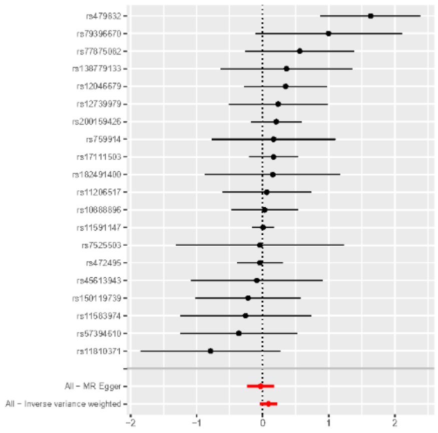

(C) inhibition of NPC1L1 on low hand grip strength

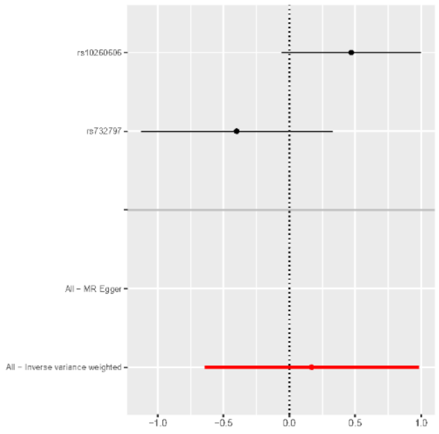

(D) inhibition of NPC1L1 on low hand grip strength*

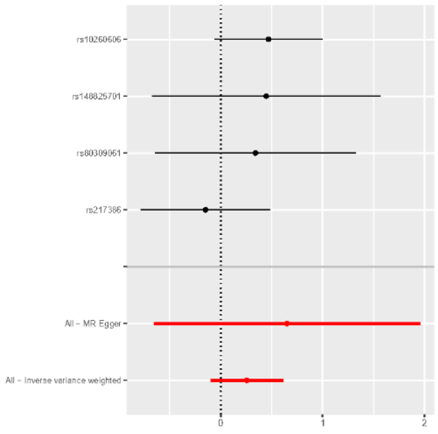

Supplementary Figure S7. Forest plots demonstrate the causal estimation of each SNP associated with inhibitions of HMGCR, PCSK9, and NPC1L1 on low hand grip strength using LDL-C from the GWAS of Sakaue et al. to proxy drug exposure. *Linkage disequilibrium (LD) threshold of r^2<0.3 in the selection of instrumental variables.

(A) inhibition of HMGCR on appendicular lean mass

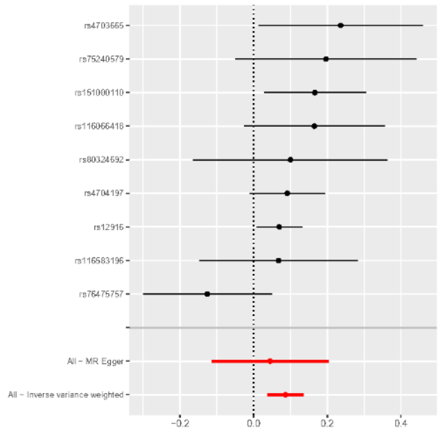

(B) inhibition of PCSK9 on appendicular lean mass

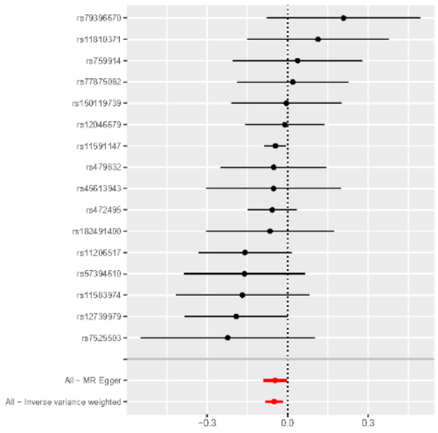

(C) inhibition of NPC1L1 on appendicular lean mass

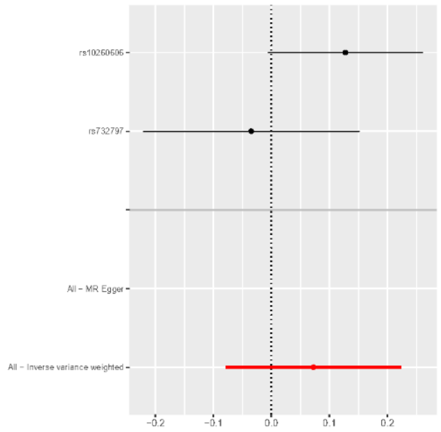

(D) inhibition of NPC1L1 on appendicular lean mass*

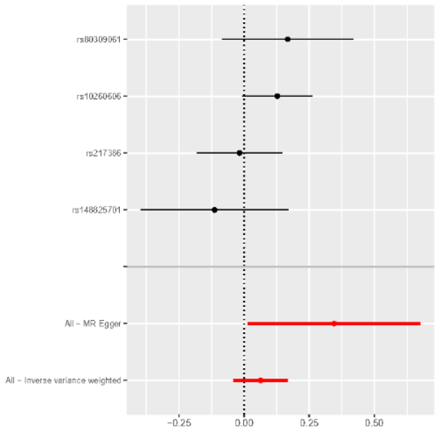

Supplementary Figure S8. Forest plots demonstrate the causal estimation of each SNP associated with inhibitions of HMGCR, PCSK9, and NPC1L1 on appendicular lean mass using LDL-C from the GWAS of Sakaue et al. to proxy drug exposure. *Linkage disequilibrium (LD) threshold of r^2<0.3 in the selection of instrumental variables.

(A) inhibition of HMGCR on usual walking pace

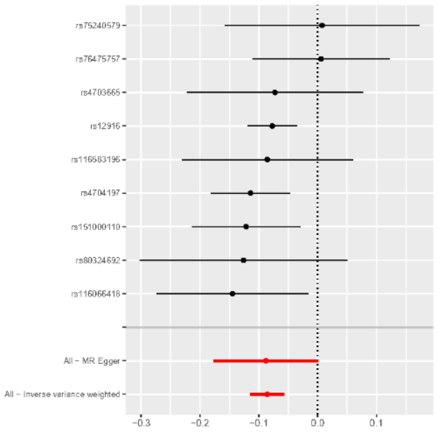

(B) inhibition of PCSK9 on usual walking pace

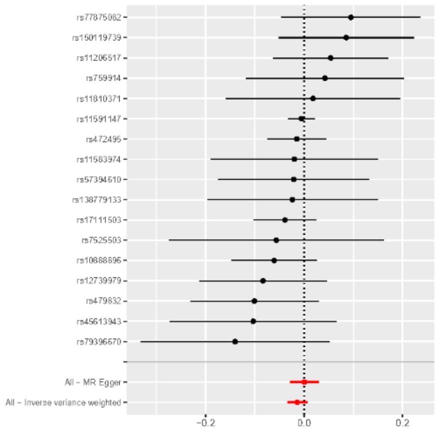

(C) inhibition of NPC1L1 on usual walking pace

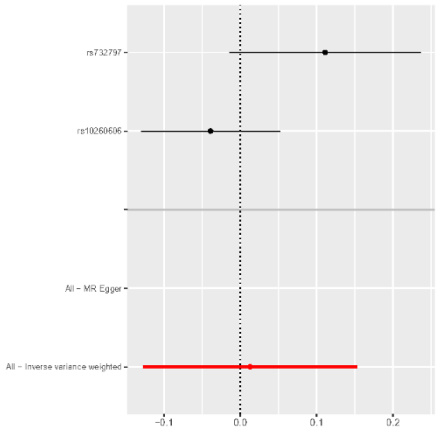

(D) inhibition of NPC1L1 on usual walking pace*

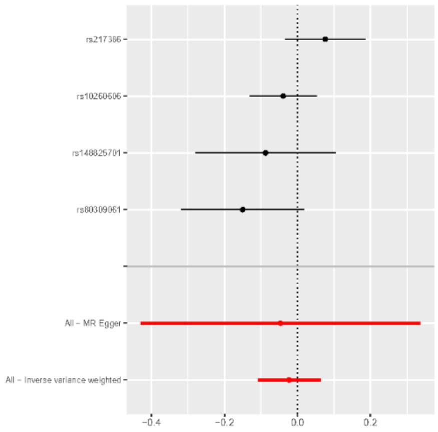

Supplementary Figure S9. Forest plots demonstrate the causal estimation of each SNP associated with inhibitions of HMGCR, PCSK9, and NPC1L1 on usual walking pace using LDL-C from the GWAS of Sakaue et al. to proxy drug exposure. *Linkage disequilibrium (LD) threshold of $r^2<0.3$ in the selection of instrumental variables.

(A) inhibition of HMGCR on low hand grip strength

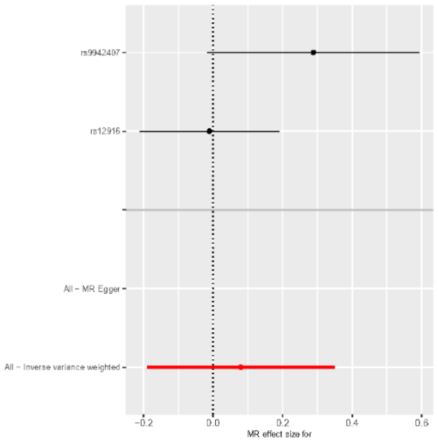

(B) inhibition of PCSK9 on low hand grip strength

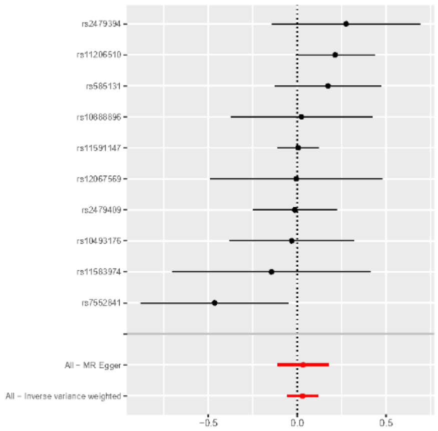

(C) inhibition of NPC1L1 on low hand grip strength

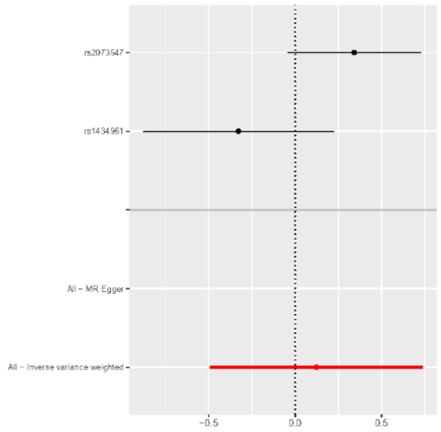

(D) inhibition of NPC1L1 on low hand grip strength*

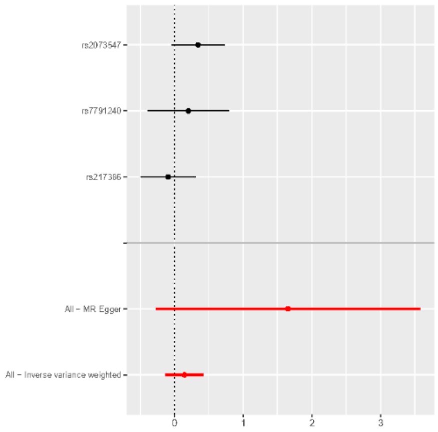

Supplementary Figure S10. Forest plots demonstrate the causal estimation of each SNP associated with inhibitions of HMGCR, PCSK9, and NPC1L1 on the low hand grip strength using LDL-C from the GLGC to proxy drug exposure. *Linkage disequilibrium (LD) threshold of $r^2<0.3$ in the selection of instrumental variables.

(A) inhibition of HMGCR on appendicular lean mass

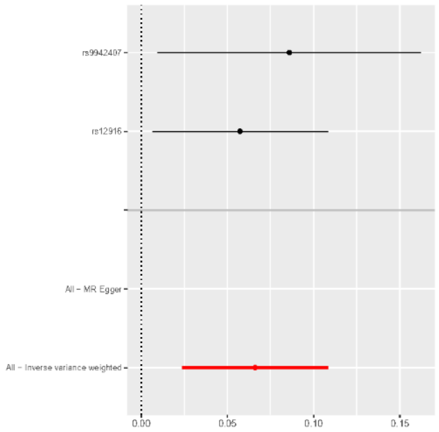

(B) inhibition of PCSK9 on appendicular lean mass

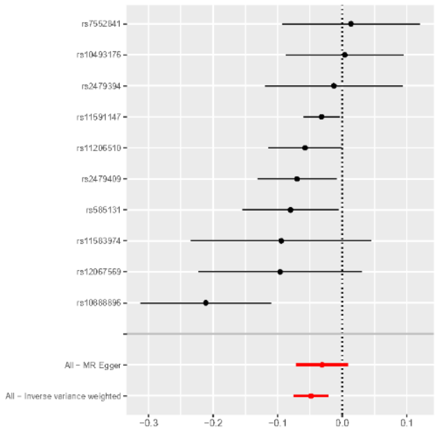

(C) inhibition of NPC1L1 on appendicular lean mass

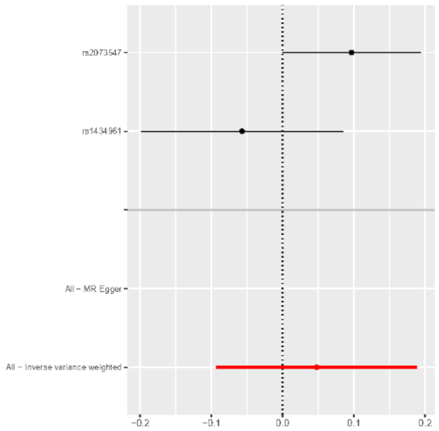

(D) inhibition of NPC1L1 on appendicular lean mass*

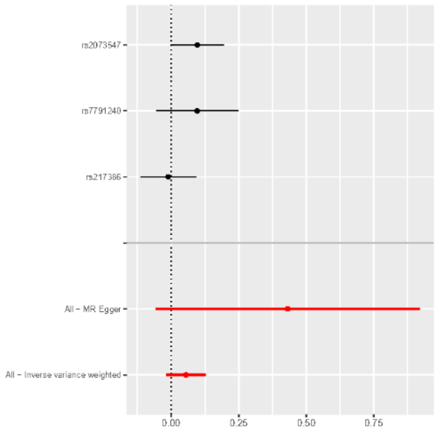

Supplementary Figure S11. Forest plots demonstrate the causal estimation of each SNP associated with inhibitions of HMGCR, PCSK9, and NPC1L1 on appendicular lean mass using LDL-C from the GLGC to proxy drug exposure. *Linkage disequilibrium (LD) threshold of $r^2<0.3$ in the selection of instrumental variables.

(A) inhibition of HMGCR on usual walking pace

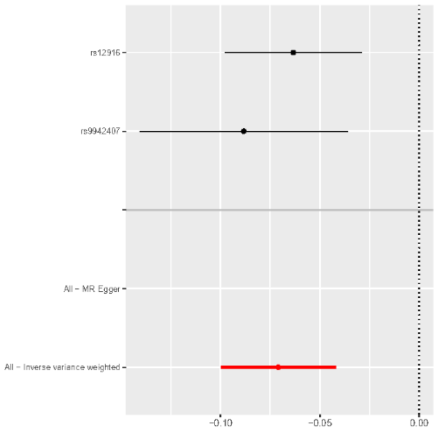

(B) inhibition of PCSK9 on usual walking pace

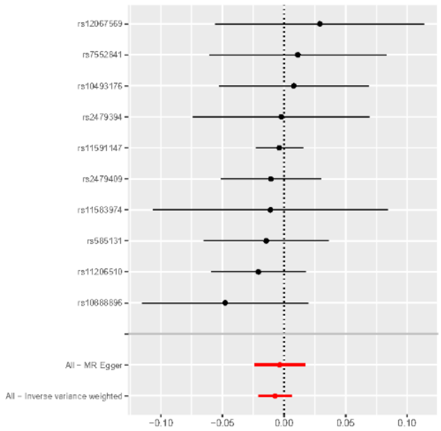

(C) inhibition of NPC1L1 on usual walking pace

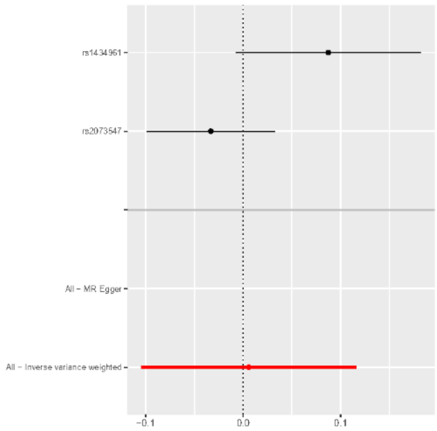

(D) inhibition of NPC1L1 on usual walking pace*

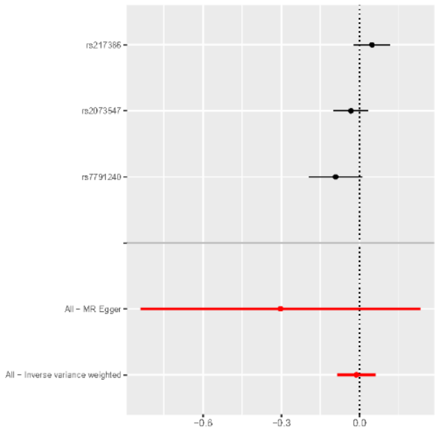

Supplementary Figure S12. Forest plots demonstrate the causal estimation of each SNP associated with inhibitions of HMGCR, PCSK9, and NPC1L1 on usual walking pace using LDL-C from the GLGC to proxy drug exposure. *Linkage disequilibrium (LD) threshold of r^2<0.3 in the selection of instrumental variables.

(A) inhibition of HMGCR on low hand grip strength

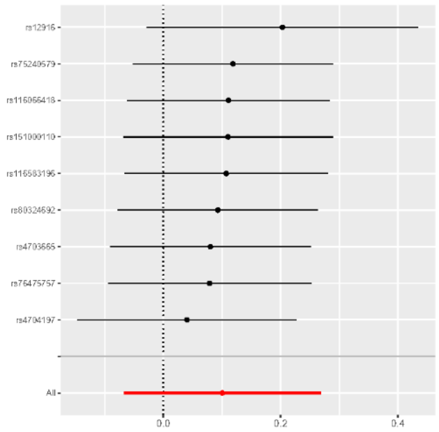

(B) inhibition of PCSK9 on low hand grip strength

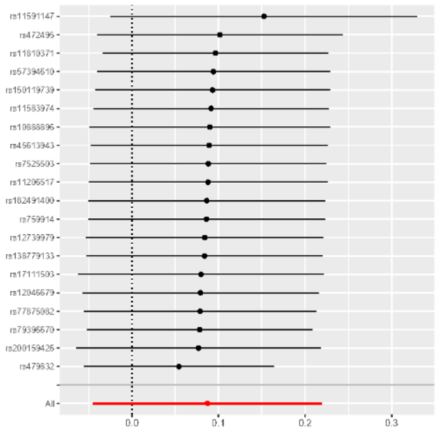

(C) inhibition of NPC1L1 on low hand grip strength*

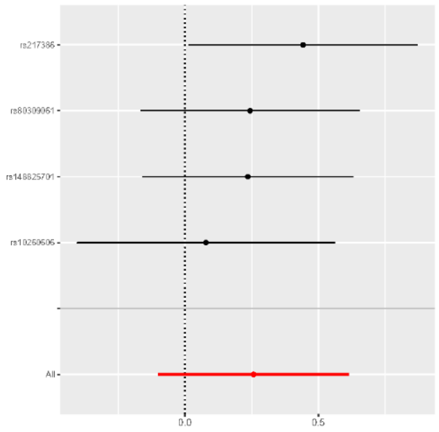

Supplementary Figure S13. Leave-one-out analysis for the causal estimation of inhibitions of HMGCR, PCSK9, and NPC1L1 on low hand grip strength using LDL-C from the GWAS of Sakaue et al. to proxy drug exposure. *Linkage disequilibrium (LD) threshold of r^2<0.3 in the selection of instrumental variables.

.

(A) inhibition of HMGCR on appendicular lean mass

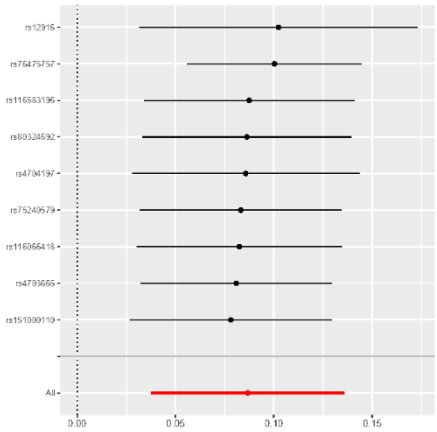

(B) inhibition of PCSK9 on appendicular lean mass

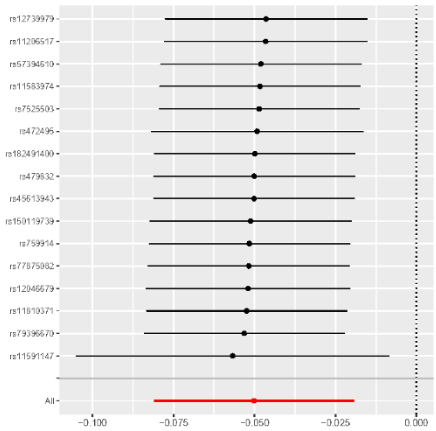

(C) inhibition of NPC1L1 on appendicular lean mass*

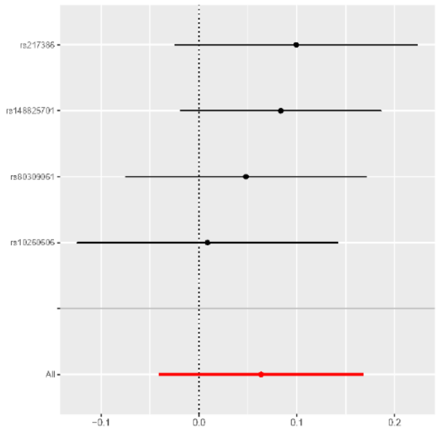

Supplementary Figure S14. Leave-one-out analysis for the causal estimation of inhibitions of HMGCR, PCSK9, and NPC1L1 on appendicular lean mass using LDL-C from the GWAS of Sakaue et al. to proxy drug exposure. *Linkage disequilibrium (LD) threshold of r^2<0.3 in the selection of instrumental variables.

(A) inhibition of HMGCR on usual walking pace

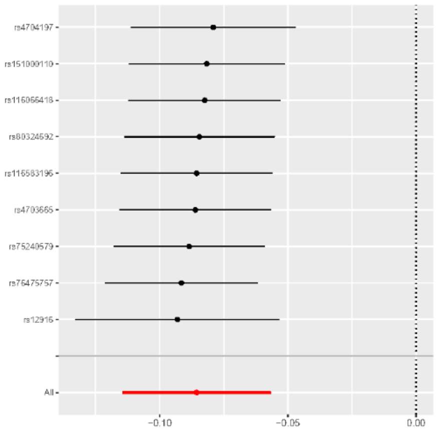

(B) inhibition of PCSK9 on usual walking pace

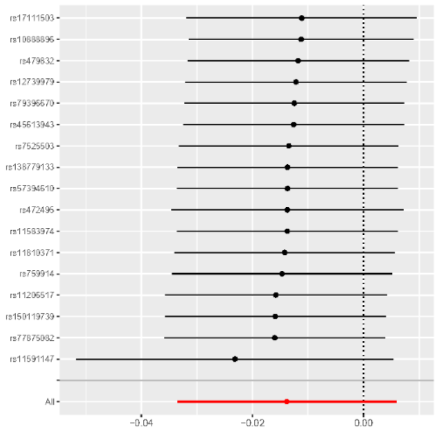

(C) inhibition of NPC1L1 on usual walking pace*

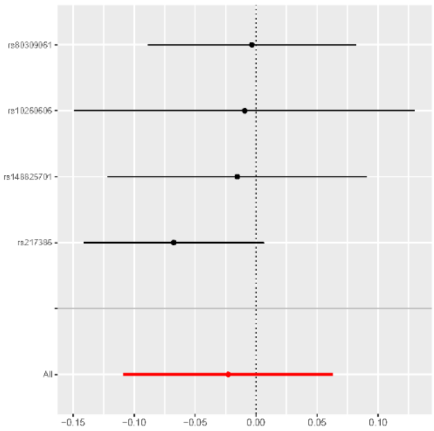

Supplementary Figure S15. Leave-one-out analysis for the causal estimation of inhibitions of HMGCR and PCSK9 on usual walking pace using LDL-C from the GWAS of Sakaue et al. to proxy drug exposure. *Linkage disequilibrium (LD) threshold of $r^2<0.3$ in the selection of instrumental variables.

(A) inhibition of PCSK9 on low hand grip strength

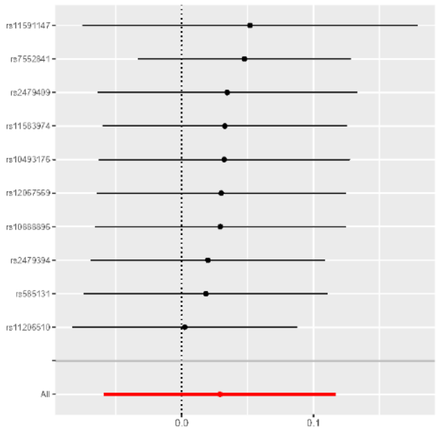

(B) inhibition of NPC1L1 on low hand grip strength*

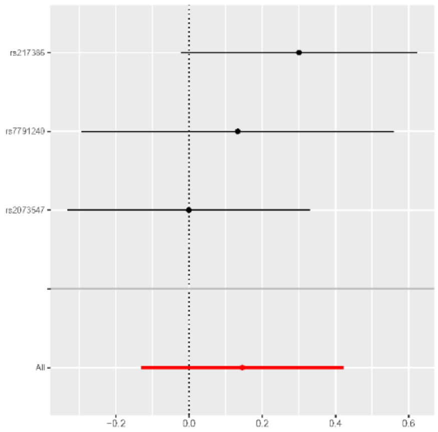

(C) inhibition of PCSK9 on appendicular lean mass

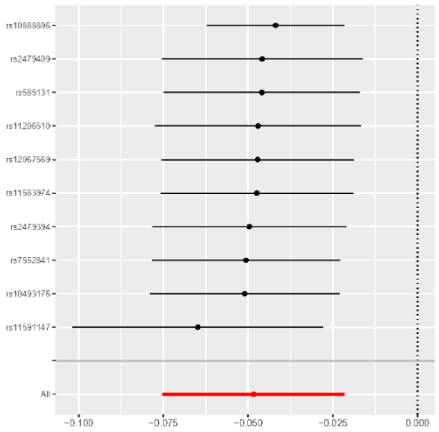

(D) inhibition of NPC1L1 on appendicular lean mass*

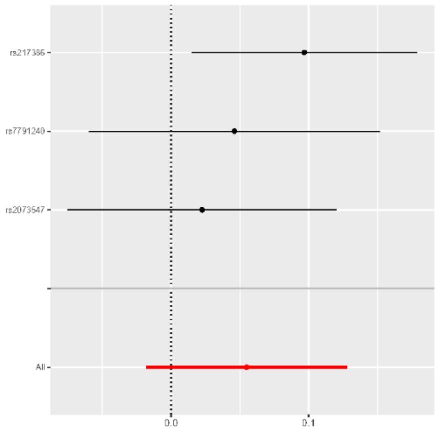

(E) inhibition of PCSK9 on usual walking pace

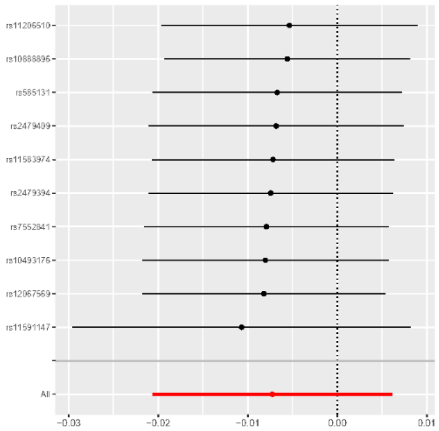

(F) inhibition of NPC1L1 on usual walking pace*

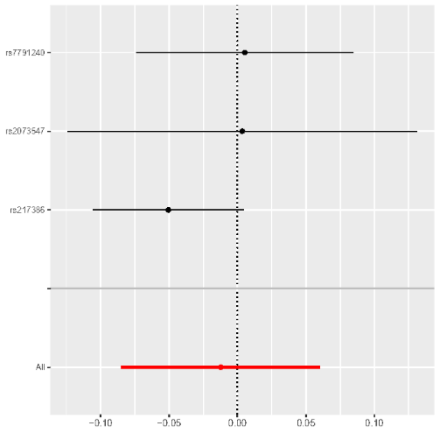

Supplementary Figure S16. Leave-one-out analysis for the causal estimation of inhibition PCSK9 and NPC1L1 on low hand grip strength, appendicular lean mass, and usual walking pace using LDL-C from the GLGC to proxy drug exposure. *Linkage disequilibrium (LD) threshold of $r^2<0.3$ in the selection of instrumental variables.

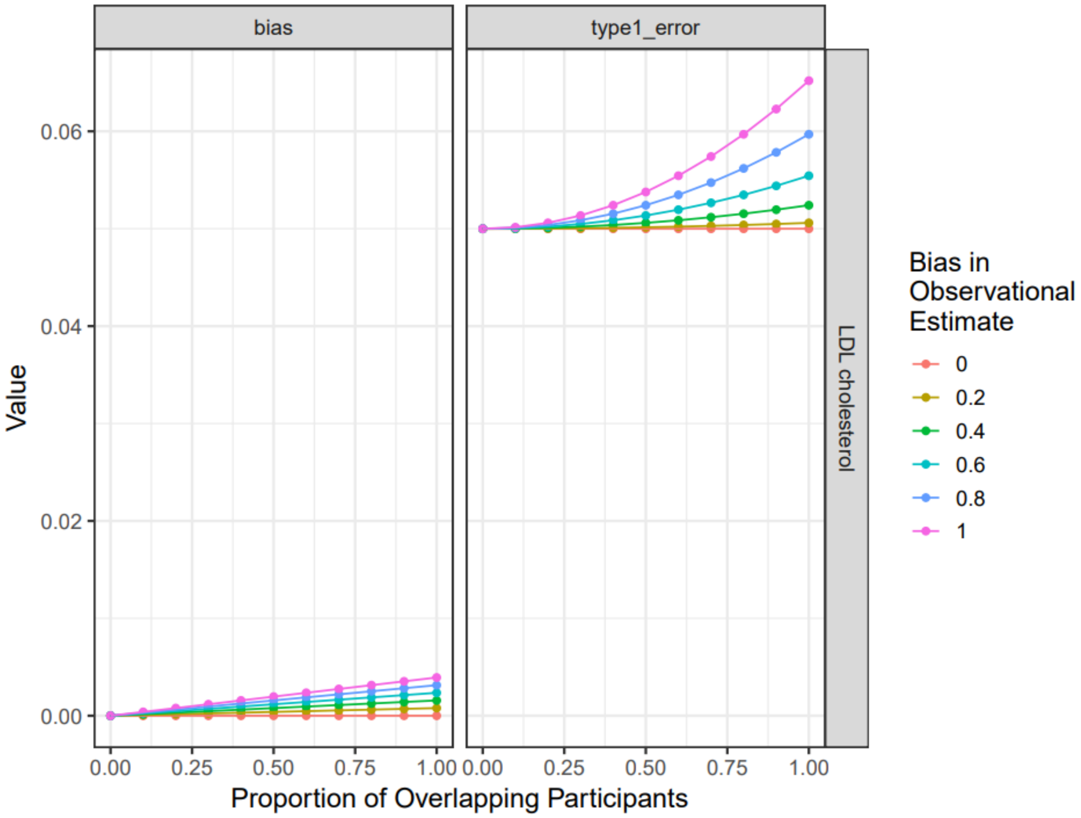

Supplementary Figure S17. The influence of sample overlap on the bias and type 1 error of Mendelian randomization analysis between the datasets of low-density lipoprotein (Sakaue et al.) and appendicular lean mass (UK Biobank).

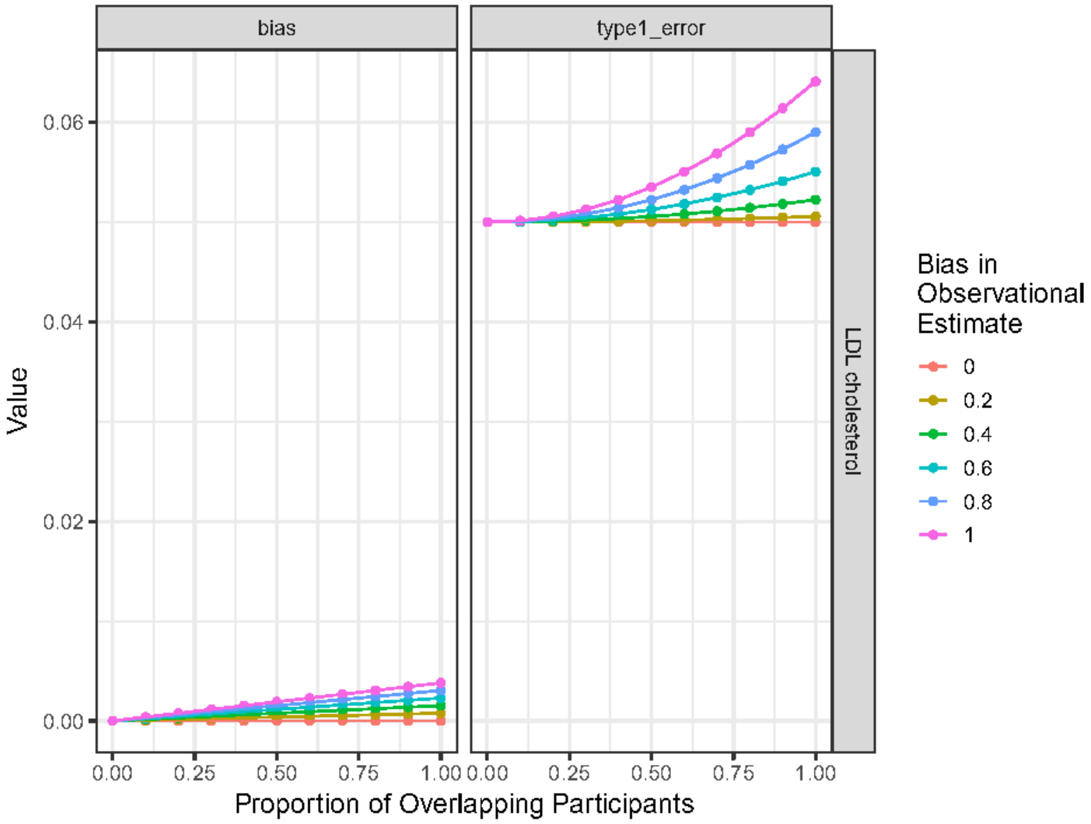

Supplementary Figure S18. The influence of sample overlap on the bias and type 1 error of Mendelian randomization analysis between the datasets of low-density lipoprotein (Sakaue et al.) and usual walking pace (UK Biobank).
